# Metabolomic Response of the Creeping Wood Sorrel *Oxalis corniculata* to Low-Dose Radiation Exposure from Fukushima’s Contaminated Soil

**DOI:** 10.3390/life11090990

**Published:** 2021-09-20

**Authors:** Ko Sakauchi, Wataru Taira, Joji M. Otaki

**Affiliations:** 1 The BCPH Unit of Molecular Physiology, Department of Chemistry, Biology and Marine Science, Faculty of Science, University of the Ryukyus, Okinawa 903-0213, Japan; yamatoshijimi@sm1044.skr.u-ryukyu.ac.jp (K.S.); wataira@lab.u-ryukyu.ac.jp (W.T.); 2 Center for Research Advancement and Collaboration, University of the Ryukyus, Okinawa 903-0213, Japan

**Keywords:** metabolome, GC-MS, LC-MS, Fukushima nuclear accident, low-dose radiation exposure, plant physiology, *Oxalis corniculata*, radioactive pollution, field effect, alfuzosin

## Abstract

The biological consequences of the Fukushima nuclear accident have been intensively studied using the pale grass blue butterfly *Zizeeria maha* and its host plant, the creeping wood sorrel *Oxalis corniculata*. Here, we performed metabolomic analyses of *Oxalis* leaves from Okinawa to examine the plant metabolites that were upregulated or downregulated in response to low-dose radiation exposure from Fukushima’s contaminated soil. The cumulative dose of radiation to the plants was 5.7 mGy (34 μGy/h for 7 days). The GC-MS analysis revealed a systematic tendency of downregulation among the metabolites, some of which were annotated as caproic acid, nonanoic acid, azelaic acid, and oleic acid. Others were annotated as fructose, glucose, and citric acid, involved in the carbohydrate metabolic pathways. Notably, the peak annotated as lauric acid was upregulated. In contrast, the LC-MS analysis detected many upregulated metabolites, some of which were annotated as either antioxidants or stress-related chemicals involved in defense pathways. Among them, only three metabolite peaks had a single annotation, one of which was alfuzosin, an antagonist of the α_1_-adrenergic receptor. We conclude that this *Oxalis* plant responded metabolically to low-dose radiation exposure from Fukushima’s contaminated soil, which may mediate the ecological “field effects” of the developmental deterioration of butterflies in Fukushima.

## 1. Introduction

Radioactive pollution caused by anthropogenic radionuclides has been widespread worldwide since the middle of the twentieth century. At present, anthropogenic ^137^Cs can be detected globally [1,2,3,4]. One of the most severe pollution events was the Fukushima nuclear accident in 2011, which was the second-largest nuclear accident after the Chernobyl nuclear accident in 1986. The biological impacts of the Fukushima nuclear accident have been studied in contaminated fields, which have focused on many organisms, including birds such as the barn swallow and goshawk [5,6,7], Japanese monkeys [8,9,10], intertidal invertebrates [11], gall-forming aphids [12,13], and plants [14,15,16,17,18]. However, to the best of our knowledge, the most intensively studied species in both field and laboratory experiments is the pale grass blue butterfly *Zizeeria maha* [19,20,21,22,23,24,25,26,27,28,29,30,31,32,33,34,35,36,37,38]. This small butterfly is popular in Japan (except for in Hokkaido) and has been established as an excellent field indicator species for environmental assessments and evolutionary studies [39,40,41] and as an excellent model organism in the laboratory for developmental and physiological studies [42,43,44,45]. Collectively, these studies have concluded that the butterflies in Fukushima have been affected both genetically by high-level initial exposure and physiologically by low-level chronic exposure through “field effects”, even though this butterfly is resistant to low-level radiation exposure from ingested ^137^Cs under experimental conditions; that is, this butterfly is dosimetrically resistant in the laboratory but vulnerable in the field, possibly due to ecological interactions.

What kind of ecological interactions in the field does this butterfly species have? The pale grass blue butterfly likely has relatively simple ecological interactions, because its life history is simple. Its larvae monophagously eat the leaves of the creeping wood sorrel *Oxalis corniculata* [20,21,42]. Adult butterflies fly around this plant and do not travel over long distances from their original location unless they are blown by a strong wind [41]. We therefore hypothesized that this host plant may change its biochemical contents after irradiation stress, even at a low level of exposure, which could then affect the butterfly larvae [27,31,32,37]. This field effect hypothesis is reasonable, considering that some plants have shown morphological and gene expression changes in response to radiation exposure due to the Fukushima nuclear accident [14,15,16,17,18]. Along this line, we discovered that the sodium content in the leaves of the host plant was inversely correlated with both the ^137^Cs radioactivity concentration in the leaves and the ground radiation dose [38]. Since the sodium deficiency in herbivore animals generally results in serious pathological consequences, this could be a possible mechanism of the ecological field effect on these butterflies. However, there may be multiple plant-mediated pathways that affect the larval physiology.

In this study, we performed metabolomic analyses of irradiated and nonirradiated *Oxalis* plants using both GC-MS (gas chromatography-mass spectrometry) and LC-MS (liquid chromatography-mass spectrometry) analyses to identify candidate metabolites with changed levels in the leaves upon irradiation. GC-MS can analyze gaseous or volatile compounds with relatively small molecular weights and relatively high heat resistance, including carbohydrates, amino acids, organic acids, and fatty acids, whereas LC–MS can analyze a wide range of compounds that can dissolve in solvents, including aromatic glycosides, terpenoid derivatives, and amino acid derivatives. Many plant primary metabolites belong to the former group of compounds, whereas many plant secondary metabolites belong to the latter. In this study, targeted and nontargeted GC-MS analyses were treated separately, because they detected two different sets of metabolite peaks.

Radiation metabolomics has often been employed to diagnostically or therapeutically search for relevant biochemicals after exposure to ionizing radiation [46,47,48,49,50]. Metabolomics was used to investigate metabolite changes in rice seeds [15,18] and calf blood plasma [51] after the Fukushima nuclear accident. To demonstrate that the creeping wood sorrel responds to the low dose of radiation that is relevant in the field, we used contaminated soil collected from Fukushima as the radiation source. For this irradiation experiment, we used whole plants collected in the field from Okinawa, the least contaminated prefecture in Japan. In this way, we reproduced in our laboratory (in Okinawa) the possible acute radioactive environment of this plant in Fukushima immediately after the nuclear accident, although the present system focused only on external exposure.

## 2. Materials and Methods

### 2.1. Plant and Culture Soil

**Figure 1:**
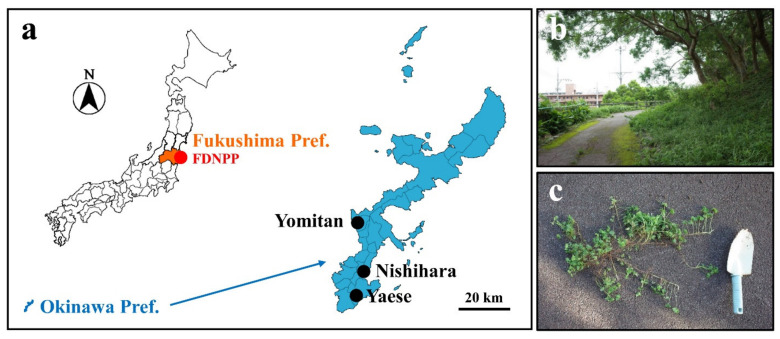
Sampling localities. (**a**) Maps of Japan and Okinawa. Shown are Fukushima Prefecture in orange (left); Fukushima Dai-ichi Nuclear Power Plant (FDNPP) in the red circle (left); Okinawa Prefecture in blue (left and right); and the 3 sampling localities (Yomitan Village, Nishihara Town, and Yaese Town) in Okinawa in black circles (right). (**b**) A landscape of the sampling site in Nishihara Town. (**c**) The creeping wood sorrel *O. corniculata* with its creeping stem just obtained at a sampling site in Yaese Town.

**Figure 2:**
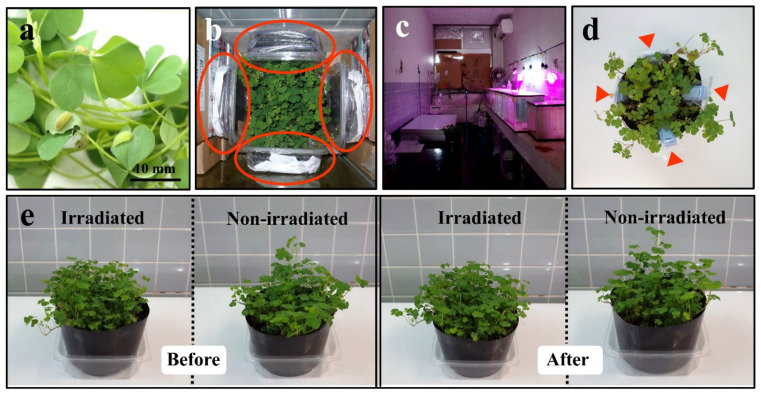
Irradiation treatment. (**a**) Typical green variant of *Oxalis* leaves used in this experiment. Larvae of the pale grass blue butterflies are also pictured (but the larvae were not irradiated in this experiment). (**b**) Top-down view of the irradiation setup. A plant pot was placed at the center, surrounded by 4 packages of contaminated soil as the radiation sources (circled). (**c**) Overview of the irradiation room. The irradiation system was set up under horticulture lights on the right and surrounded by aquarium tanks filled with water. (**d**) Four dosemeters in a pot (arrowheads). (**e**) Irradiated and nonirradiated *Oxalis* plants before and after treatment.

Whole plants of the creeping wood sorrel *O. corniculata,* including the roots, were obtained from 3 localities on Okinawa-jima Island, Okinawa Prefecture, Japan: Nishihara Town (Uehara-Takadai Park), Yomitan Village (near Zakimi Castle Ruin), and Yaese Town (Kochinda Undo Park) (Figure 1). This plant has color variants, but a typical green variant was used (Figure 2a). To generate a pair of genetically identical samples, each individual plant was separated into two batches with the same identification number (1: Yaese, 2: Nishihara, 3: Yomitan). That is, a pair of two batches were a clone. The clones were potted individually in cylindrical pots (100 mm in diameter × 150 mm in height) using a package of commercially available culture soil for flowers and vegetables, Hanasaki Monogatari (Akimoto Tensanbutsu, Iga, Mie, Japan). This soil was analyzed for its radioactivity concentration in a way similar to that used for the contaminated soil from Fukushima (see below). We detected 2.14 Bq/kg ^137^Cs (*n* = 1; measured on 8 August, 2018), but we considered this amount to be negligible in comparison to the contaminated soil from Fukushima (see below). The potted plants were grown outside under natural conditions for approximately 2 weeks until many leaves were produced. The plants were watered every day before and during irradiation treatment.

### 2.2. Irradiation Treatment

Plants of the experimental group (irradiated; IR) were treated with external radiation for 7 days (168.0 h) at room temperature (27 °C ± 1 °C) (Figure 2b), and plants of the control group (nonirradiated control; NC) were placed under the same conditions but without irradiation. To do so, plants were kept under 18 L:6 D-long day conditions using Derlights horticulture LED light bulbs (40-W equivalence) (Figure 2c). The plants in the irradiation group were surrounded by 4 transparent plastic square containers (135 mm × 135 mm × 57 mm) containing contaminated soil, which were then surrounded by a concrete wall, two walls of several lead blocks (a single block: 100 mm × 200 mm × 50 mm), and aquarium tanks filled with water (Figure 2b). The water tanks and lead blocks were further aligned to minimize the radiation exposure of the researchers (Figure 2c). The plants in the nonirradiated control group were surrounded similarly but without the soil containers. The irradiation periods for the Nishihara, Yomitan, and Yaese plant samples were 25 September–2 October, 8–15 October, and 18–25 November 2018, respectively.

Contaminated soil as the plant radiation source described above was collected in Minamisoma City, Fukushima Prefecture on 28 November 2014 after an evacuation order for that area was partially lifted. Surface soil at a depth of 0–50 mm was collected. The soil was packed in plastic bags, which were then contained in transparent plastic square containers, as described before. For evaluation of the cumulative absorbed dose under these conditions, 4 dosemeters were put in a pot (see below) (Figure 2d).

We confirmed that the plants looked equally healthy before and after treatment; no signs of leaf necrosis, chlorosis, or other abnormalities were found by visual inspection (Figure 2e). After the 7-day exposure period, the plant leaves (5–10 g per plant sample) were handpicked with disposable gloves. Relatively young leaves were preferably collected, and relatively old ones were excluded, because young leaves are likely preferred by butterfly larvae and because secondary metabolites may be more abundant in young leaves. The leaf samples were then washed with Evian bottled natural mineral water (Evian les Bains, France). After that, the water was completely drained from the leaves. The leaves were then pat-dried and quickly frozen in liquid nitrogen. The frozen samples were packed with dry ice and sent to the Kazusa DNA Research Institute, Kisarazu, Chiba, Japan (www.kazusa.or.jp, accessed on 15 September 2021) for GC-MS and LC-MS analyses (see below).

### 2.3. Cumulative Dose and Dose Rate

To measure the cumulative dose of radiation to the plants, we used a wide-range glass badge dosemeter for the environment (type code: ES) (60 mm × 28 mm × 16 mm) from Chiyoda Technol (Tokyo, Japan). This dosemeter is the latest-generation dosemeter, which takes advantage of radiophotoluminescence (RPL). According to the manufacturer’s specifications, this dosemeter can detect X-rays (10 keV–10 MeV), γ-rays (10 keV–10 MeV), and β-rays (130 keV–3 MeV) and can measure 0.1 mSv–10 Sv of radiation. In the present study, there was no X-ray source, and the β-rays were probably shielded by the plastic containers but only to some extent. Thus, we considered that the dosemeters mainly detected γ-rays, as well as β-rays. We put each dosemeter in a plastic bag for waterproofing, and four of them were placed in a pot for 7 days (168.0 h) (Figure 2d). Two new pots were prepared from a plant collected from Nanjo City (Fusozaki Park), Okinawa: one for irradiation and the other for the nonirradiated control, as explained above. After 7 days (168.0 h) of exposure (16–23 October 2018) under the experimental conditions above, the dosemeters were sent back to Chiyoda Technol, where image development and quantification were performed. The outputs were given as dose equivalents at a depth of 70 μm from the human body surface in sieverts (Sv). We assumed that the process of dose absorption on the surface is similar between humans and *Oxalis* plants and that Sv values in humans and Gy values in plants may be interchangeable.

### 2.4. Radioactivity Concentration of the Contaminated Soil from Fukushima

The contaminated soil from Fukushima was placed in a cylindrical columnar plastic vial (15 mm in diameter and 50 mm in height) to make an 8-mm sample height, and its radioactivity concentration was measured using a Canberra GCW-4023 germanium semiconductor radiation detector (Meriden, CT, USA) until an error rate of less than 2% was reached. The counting efficiency values for ^40^K and ^137^Cs were 7.92% and 16.62%, respectively. The branching ratios for ^40^K and ^137^Cs used here were 10.67% and 85.21%, respectively. The half-lives of ^40^K and ^137^Cs used here were 1.251 × 10^9^ years and 30.17 years, respectively. Three soil samples were measured, and their outputs were averaged. The measurements were performed from 9 May to 1 July, 2016. All calculations of the radioactivity concentrations were set at the first exposure date, 25 September, 2018, for simplicity.

### 2.5. GC-MS Sample Preparation

The leaf samples were first mixed with methanol to make a 75–80% methanol solution, which was homogenized using zirconia beads. After centrifugation at 15,000 rpm for 5 min, the supernatants were collected and subjected to a MonoSpin C18 column (GL Sciences, Tokyo, Japan). The column was pretreated with 100% methanol and centrifuged at 5000× *g* for 2 min, and 70% methanol was further added. The column was again centrifuged at 5000× *g* for 2 min. The sample was then applied to this pretreated column, which was then centrifuged at 3000× *g* for 2 min. The eluted samples were collected and subsequently treated with nitrogen gas and methoxamine hydrochloride with pyridine for methoxime derivatization of the compounds and then with MSTFA (*N*-methyl-*N*-(trimethylsilyl)trifluoroacetamide) for trimethylsilyl (TMS) derivatization.

### 2.6. GC-MS Analysis

The samples were analyzed using a SHIMADZU gas chromatograph quadrupole mass spectrometer GCMS-QP2010 Ultra (Kyoto, Japan) with a SHIMADZU autosampler AOC-5000 Plus and an Agilent Technologies DB-5 column (30 m in length, 0.250 mm in internal diameter, and 1.00 μm in membrane thickness) (Santa Clara, CA, USA). The electron ionization (EI) method was used to ionize the samples. The following setting values were employed: chamber temperature, 280 °C; oven temperature, 100 °C for 4 min at a rate of 4 °C/min, followed by holding at 320 °C for 8 min; connection temperature, 280 °C; ionization source temperature, 200 °C; flow rate, 390 mm/s (1.1 mL/min); scanning speed, 2000 u/s; mass detection range, 45–600 (*m/z*); and sample injection volume, 0.5 μL. An autotuning function was used for machine tuning and validation, in which a standard calibration sample of PFTBA (perfluorotributylamine) was used for tuning the resolution, sensitivity, mass calibration, and vacuum. For time adjustment, mixed alkanes C_7_–C_33_ were analyzed. An internal standard was not used.

### 2.7. GC-MS Peak Detection, Alignment, and Annotation

The targeted and nontargeted (untargeted) methods were performed independently. For the targeted methods, SHIMADZU analysis software GCMSsolution and its associated GC-MS Metabolite Database ver. 2 were employed for compound annotation and comparisons between samples. Peaks were detected in reference to those in the database based on specific fragment ions and retention times and were annotated based on the following three points between the peaks detected in the sample and those in the compound library: similarity in the mass spectral patterns, similarity in the intensity ratios of the specific mass fragments, and similarity in the retention times. When two or more peaks were annotated as the same chemical compound, the area values and retention time values of those peaks were extracted and aligned.

For the nontargeted methods (analysis of all peaks), SpectraWorks MS spectrum data mining software AnalyzerPro (Runcorn, Cheshire, UK) and SHIMADZU GC-MS peak alignment software FragmentAlign (www.kazusa.or.jp/komics/software/FragmentAlign, accessed on 15 September 2021) [52] were employed. Peaks were detected automatically by AnalyzerPro based on the peak area, peak width, signal-to-noise ratio, and specific thresholds. Whether the ions originated from the same chemical compound was examined by a deconvolution treatment based on the peak retention time and peak shape. When it was concluded that the ions had an identical origin, they were bundled together. Alignments were performed based on the scores (using Pearson product-moment correlation and cosine similarity) and retention times. Chemical compound annotations were determined based on the similarity between the mean mass spectrum patterns of the samples and those of the chemical compound library. The results of both the targeted and nontargeted methods were compiled in a Microsoft Excel file, “GC-MS_Result Kazusa DNA Institute” (Supplementary Table S1).

### 2.8. LC-MS Sample Preparation

The leaf samples were put in methanol (final concentration of 75%), homogenized with zirconia beads, and centrifuged at 15,000 rpm for 10 min. The supernatant was collected. Additionally, a MonoSpin C18 column (GL Sciences) was treated with 100% methanol and centrifuged at 5000× *g* for 2 min. The column was again treated with 75% methanol and centrifuged at 5000× *g* for 2 min. The prepared sample was then applied to the column, which was centrifuged at 5000× *g* for 2 min. The eluted solution was collected and filtered through a 0.2-μm filter. Each sample was analyzed 3 times for reproducibility, and the results were averaged before the statistical analyses.

### 2.9. LC-MS Analysis

The samples were analyzed using a SHIMADZU Nexera X2 high-performance liquid chromatography (HPLC) instrument connected to a Thermo Fisher Scientific Q Exactive Plus high-resolution mass analyzer (Waltham, MA, USA). An InertSustain AQ-C18 column (2.1 mm × 150 mm, 3-μm particle size) (GL Sciences) was used as the reversed-phase HPLC column. A Nexera X2 system was run under the following conditions: column temperature, 40 °C; mobile phase A, 0.1% formic acid in water; mobile phase B, acetonitrile; flow of mobile phase, 0.2 mL/min; and sample injection volume, 2 μL. The Q Exactive Plus instrument was run under the following conditions using electrospray ionization (ESI): measurement time, 3–30 min; measurement range, 80–1200 (*m/z*); full-scan resolution, 70,000; and MS/MS scan resolution, 17,500. For MS/MS precursor selection, a Data-Dependent Scan (Top 10) was performed, in which the top 10 precursor ions detected by the full scan were subjected to MS/MS analysis. The dynamic exclusion was 20 s, in which the precursor ions that were previously measured were excluded from the MS/MS analysis to measure as many precursor ions as possible.

An LCMS QC Reference Material (Waters, Milford, MA, USA) containing 9 known standard chemical compounds with known concentrations was analyzed before and after the sample analysis to confirm that the retention times and detection sensitivity were set within acceptable ranges. The maximum ion intensity from each sample was confirmed to be sufficient for the peak signal detection (10^8^–10^9^ cps). The LCMS QC Reference Material contained the following compounds: acetaminophen (C_8_H_9_NO_2_) at 152.0712 (10 μg/mL), caffeine (C_8_H_10_N_4_O_2_) at 195.0882 (1.5 μg/mL), sulphaguanidine (C_7_H_10_N_4_O_2_S) at 215.0603 (5 μg/mL), sulfadimethoxine (C_12_H_14_N_4_O_4_S) at 311.0814 (1 μg/mL), Val-Tyr-Val (C_19_H_29_N_3_O_5_) at 380.2185 (2.5 μg/mL), verapamil (C_27_H_38_N_2_O_4_) at 455.2910 (0.2 μg/mL), terfenadine (C_32_H_41_NO_2_) at 472.3216 (0.2 μg/mL), Leu-enkephalin (C_28_H_37_N_5_O_7_) at 556.2771 (2.5 μg/mL), and reserpine (C_33_H_40_N_2_O_9_) at 609.2812 (0.6 μg/mL).

### 2.10. LC-MS Peak Detection, Alignment, and Annotation

The LC-MS data obtained above were converted to mzXML format using ProteoWizard (http://proteowizard.sourceforge.net, accessed on 15 September 2021). Peak detection, determination of the ionizing states, and peak alignments were performed automatically using the data analysis software PowerGetBatch (http://www.kazusa.or.jp/komics/software/PowerGetBatch/ja, accessed on 15 September 2021) developed by the Kazusa DNA Research Institute [53]. Each detected peak was associated with a retention time, a peak area (intensity), an exact mass, and a MS/MS spectrogram. At this point, a collection of detected peaks included not only those from compounds within the samples but, also, those from noise and false positives. This is partly because a single compound is detected as multiple peaks, depending on the ionization state after ESI. Thus, the ionization states (adducts) were determined based on differences in the exact masses of the peaks detected with similar retention times. Each pair of peaks that was consistent with the known adduct pairs was identified based on the assumption that the basic cation adduct was [M+H]^+^ and the basic anion adduct was [M-H]^−^. When pairing was not possible, either [M+H]^+^ or [M-H]^−^ was assigned, because these ions were produced most frequently. All the detected peaks were aligned using all samples based on their *m/z* and retention time values, resulting in a peak intensity matrix. The noise peaks and false-positive peaks were then eliminated based on the negative controls. The detected peaks were considered valid when they were reproducibly detected in each of three trials of an identical sample.

Exact mass values of the nonionized compounds calculated from the adducts were searched against the following chemical mass databases developed by the Kazusa DNA Research Institute: (1) UC2 (http://webs2.kazusa.or.jp/mfsearcher/uc2/, accessed on 15 September 2021) [54], which contains metabolites recorded in the following two databases: KNApSAcK (http://knapsackfamily.com/KNApSAcK_Family/, accessed on 15 September 2021) [55] and Human Metabolome Database (https://hmdb.ca, accessed on 15 September 2021), (2) a theoretical chemical composition formula database (containing chemically reasonable compounds and peptides less than molecular weight 1000), and (3) an in-house database. The database searches were performed using the search program MFSearcher [56] developed by the Kazusa DNA Research Institute. When the alignments had a compound that matched its own mass (±1 ppm) from these databases, a chemical composition formula was assigned. When a matching compound was not found within ±5 ppm of its own mass, its mass was adjusted by adding ±1 ppm, and a new search was performed.

Valid alignments (peaks) were further examined against the reference material standards that were analyzed previously under the same conditions at the Kazusa DNA Research Institute. The reference standards included plant metabolite data that were provided by the collaborative study between the Kazusa DNA Research Institute and Tokiwa Phytochemical (Sakura, Chiba, Japan). The mean *m/z* values and MS/MS spectrograms of a set of alignments were compared with those of reference material standards. Within a mean retention time ±1.5 min, the cosine similarity of the MS/MS spectrograms must be >0.9, and the *m/z* error must be within ±5 ppm to be considered likely identical. The LC-MS/MS results were compiled in the Microsoft Excel file “LC-MS_Result Kazusa DNA Research Institute” (Supplementary Table S2).

### 2.11. Statistical Analysis of the Peak Area Data

The output peak data of GC-MS were compiled in Microsoft Excel files (Supplementary Table S3 and S4). The output peak data of LC-MS were also treated similarly (Supplementary Table S5). The peak area (intensity) data were subjected to statistical analyses using MetaboAnalyst 5.0 [57,58,59]. In the process of uploading the data into MetaboAnalyst, the peak data with a constant or single value across the samples were deleted automatically. The data were subjected to autoscaling, a mean-centered normalization process divided by the standard deviation of each sample. However, box plots and graphs related to the *t*-test and fold change analysis were made using the original peak area values without normalization to understand the original levels, although the original levels did not necessarily reflect the levels in the leaf samples in these analyses. During upload, data filtering was not performed. Sample normalization to adjust for differences among the samples was not performed.

We performed Student’s *t*-tests (unpaired and bi-sided) and used *p* < 0.05 (without adjustment) as the criterion to consider statistical significance, and the peaks that met this criterion were examined independently. The statistical contribution of pairs was ignored, because MetaboAnalyst cannot perform such calculations when the number of peaks exceeds 1000, and because we confirmed that the *p*-values did not change much with or without pairing. We also performed a fold change analysis and referred to fold change (*FC*) values, and peaks with *FC* > 2.0 for upregulation and *FC* < −2.0 for downregulation were considered important. When calculating the *FC* values, MetaboAnalyst uses the equations *FC* = *a*/*c* when *a* > *c* and *FC* = −*c*/*a* when *a* < *c*, where *a* is an experimental group and *c* is a control group. However, we also manually calculated *a*/*c* when *a* < *c* to examine the candidate peaks independently. In this case, *FC* < 0.5 was considered important. A principal component analysis (PCA) and heat map analysis were performed to obtain possible relationships among the samples. In the latter, Euclidean distance and the Ward linkage method were employed for clustering.

### 2.12. Comparison of the LC-MS/MS Spectrograms for Alfuzosin

To clarify the identity of peak No. 4746, we referred to public LC-MS/MS spectrogram records of alfuzosin in the Human Metabolome Database (HMDB) ver. 4.0 (https://hmdb.ca) (accessed on 21 July 2021) [60,61]. Alfuzosin (HMDB0014490) had 10 experimental [M+H]^+^ spectrograms available as of July 2021, which were referred to in this study.

## 3. Results

### 3.1. Cumulative Dose, Dose Rate, and Radioactivity Concentration

The cumulative absorbed doses after 7 days (168.0 h) from the four dosemeters were 7.1, 4.6, 5.5, and 5.5 mGy, resulting in a value of 5.7 ± 1.0 mGy (mean ± standard deviation). That is, the dose was on the order of milligrays in this system. The dose rates from four dosemeters were calculated to be 42, 27, 33, and 33 μGy/h, resulting in a value of 34 ± 6 μGy/h. In contrast, all four dosemeters in the nonirradiated control showed no detection.

The contaminated soil contained 363.54 ± 11.25 Bq/kg ^40^K and 1.529 ± 0.013 MBq/kg ^137^Cs. Since these are the major radionuclides in the soil, and because ^137^Cs outnumbered ^40^K, it is likely that the plant mostly received γ-rays and β-rays from anthropogenic ^137^Cs from the Fukushima Dai-ichi Nuclear Power Plant.

### 3.2. GC-MS: Targeted Method

**Figure 3:**
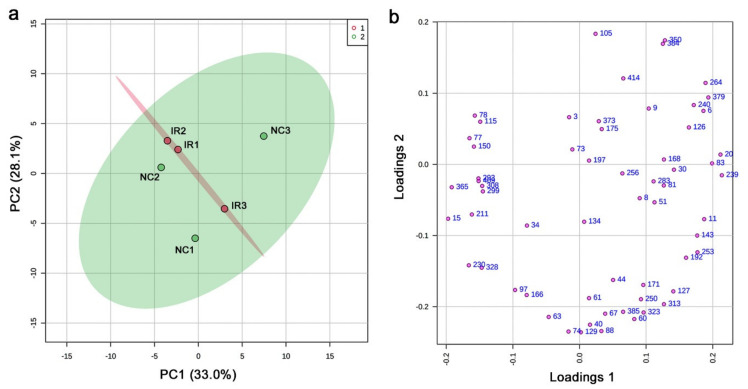
PCA results from the targeted GC-MS analysis. (**a**) Score plot; 95% confidence ranges are colored. IR, irradiated; NC, nonirradiated. (**b**) Loading plot; 61 peaks are located in a 2D plane.

In the targeted GC-MS method, 428 compounds were targeted; the shortest retention time was 7.444 min (boric acid), and the largest retention time was 62.738 min (cholesterol). Using six plant samples (three irradiated and three nonirradiated), 61 peaks were detected in total. To understand the relationships among the six plant samples, we first performed a principal component analysis (PCA) using the output peak area (intensity) data (Figure 3a). In the score plot, the nonirradiated samples were scattered, but the irradiated samples were relatively clustered together, suggesting that these plants might have responded to irradiation in a stereotypical manner despite genetic differences. However, PC1 and PC2 explained only 33.0% and 28.1% of the variation, respectively. In the loading plot, the 61 peaks were mostly scattered (Figure 3b).

**Figure 4:**
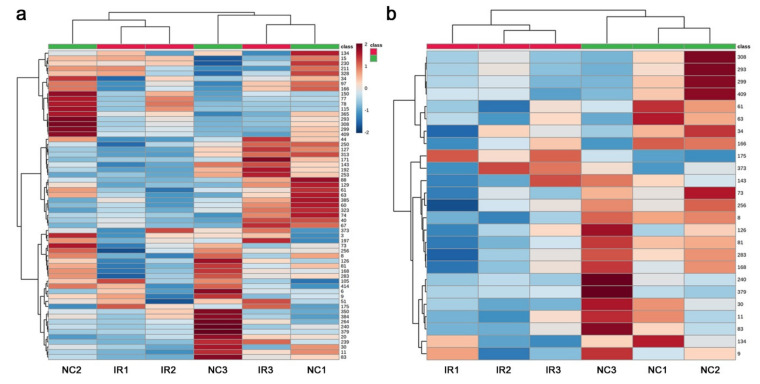
Heat maps from the targeted GC-MS analysis. At the top, the irradiated group was indicated by red horizontal bars, and the nonirradiated control group was indicated by green horizontal bars. (**a**) Heat map using all 61 peaks. (**b**) Heat map using the top 25 peaks.

A heat map using all 61 peaks revealed some overall trends. All three nonirradiated samples (NC1, NC2, and NC3) had high levels of peaks at certain locations on the map, whereas two irradiated samples (IR1 and IR2) had only relatively low levels of peaks (Figure 4a). All three nonirradiated samples had different patterns, and their corresponding irradiated samples showed different patterns from those of the nonirradiated samples. Using the top 25 peaks, the heat map clearly indicated that the irradiated and nonirradiated groups were differently clustered (Figure 4b). The three irradiated samples were similar to one another, and the three nonirradiated samples were less similar to one another. Overall, the nonirradiated samples were more diverse and robust in metabolite levels than the irradiated samples, at least as determined by the targeted GC-MS method. These results suggest that the plant responded to irradiation at the metabolome level despite the genetic differences, probably by slowing down some metabolic pathways, although the response patterns were not very consistent among the three irradiated plant samples.

**Figure 5:**
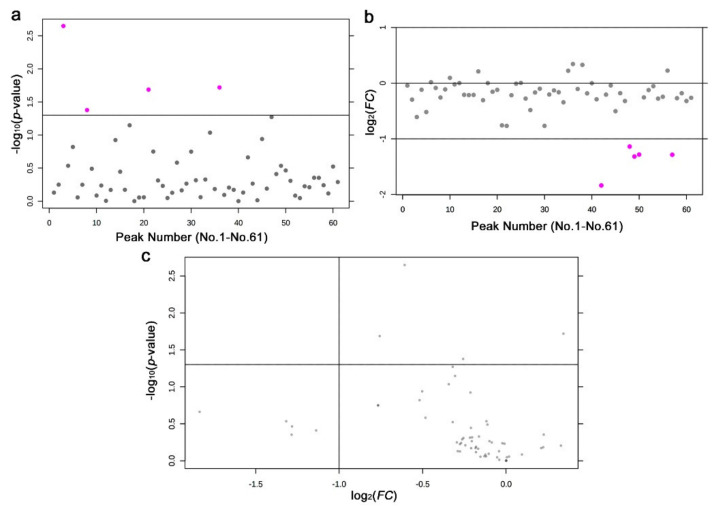
Results of the *t*-test and fold change analysis from the targeted GC-MS analysis between the irradiated and nonirradiated groups. (**a**) Distribution of the *p*-values (Student’s *t*-test). The threshold was set at *p* = 0.05. The 4 pink dots indicate significant peaks. The peak numbers (No. 1–No. 61) here on the horizontal axis do not correspond to the original peak numbers shown in Figure 6, because the original peak numbers were assigned before elimination of the nonsense peaks by MetaboAnalyst (also in (**b**)). (**b**) Distribution of the *FC* values. The thresholds were set at *FC* = 2.0 and *FC* = −2.0. The 5 pink dots indicate peaks that were downregulated more than twofold. (**c**) Volcano plot. No peak satisfied both the *p*-value and *FC* value criteria.

To examine the differences in the peak area values between the irradiated and nonirradiated groups, we performed *t*-tests and fold change analyses. We detected four peaks with significant differences (*p* < 0.05) (Figure 5a). Similarly, we detected five peaks with *FC* > 2.0 or *FC* < −2.0 (Figure 5b). Notably, most peaks had negative *FC* values, indicating that they were mostly downregulated, which was consistent with the heat map results. It was notable that, in all five cases, the distribution ranges of the peak area values were much wider in the nonirradiated samples than in the irradiated samples, suggesting a stereotypical response among the three plants irrespective of the genetic differences. There were no peaks that satisfied both the *t*-test and fold change analysis conditions (*p* < 0.05 and *FC* > 2.0 or *FC* < −2.0) (Figure 5c).

**Figure 6:**
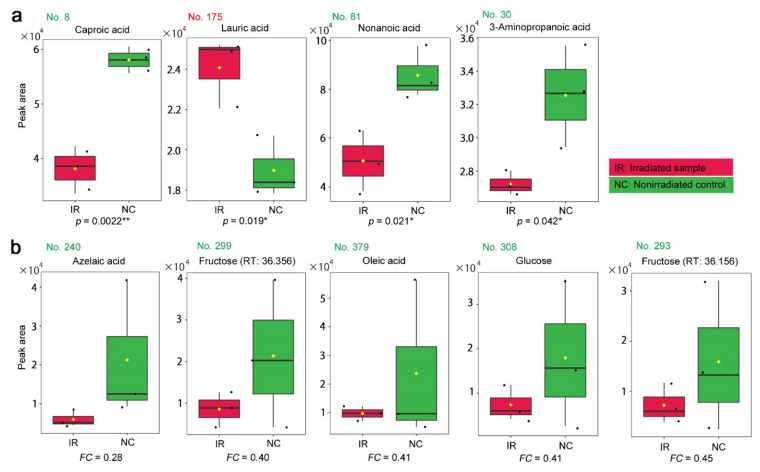
Box plots of the peak area (intensity) values from the targeted GC-MS analysis. The irradiated group (IR) is indicated by red boxes, and the nonirradiated control group (NC) is indicated by green boxes. The peak numbers are shown in red when upregulated, and they are shown in green when downregulated. (**a**) Peaks with significant differences between the irradiated and nonirradiated groups in the order of low-to-high *p*-values. There were 4 annotated peaks with *p* < 0.05 (Student’s *t*-test). (**b**) Peaks identified by a fold change analysis between the irradiated and nonirradiated groups in the order of low-to-high *FC* values. There were 5 annotated peaks with *FC* > 2.0 or *FC* < 0.5. * *p* < 0.05, ** *p* < 0.01.

The four peaks with *p* < 0.05 were annotated as caproic acid (No. 8, *p* = 0.0022), lauric acid (No. 175, *p* = 0.019), nonanoic acid (No. 81, *p* = 0.021), and 3-aminopropanoic acid (β-alanine) (No. 30, *p* = 0.042) (Figure 6a). Among them, only lauric acid was upregulated after irradiation, and the others were downregulated. Only caproic acid satisfied *p* < 0.01. The five peaks with *FC* < 0.5 (i.e., *FC* < −2.0) were annotated as azelaic acid, fructose, oleic acid, glucose, and fructose (two peaks with slightly different retention times were both annotated as fructose) (Figure 6b). These compounds were all downregulated after irradiation. The box plots indicated, in a cautionary manner, that their irradiated and nonirradiated distributions overlapped, except for in the case of azelaic acid. An outstanding single value for oleic acid in the nonirradiated group likely contributed greatly to *FC* < 0.5.

The *Oxalis* plant is known to contain a large amount of oxalic acid, as implied by the name. Oxalic acid in the leaves has been proposed to function as a feeding stimulant for larvae of the pale grass blue butterfly [62]. The abundant presence of oxalic acid in the leaves was verified as peak No. 15 in this analysis. Its peak levels were not significantly different between the irradiated and nonirradiated groups (*p* = 0.87; Student’s *t*-test).

### 3.3. GC-MS: Nontargeted Method

**Figure 7:**
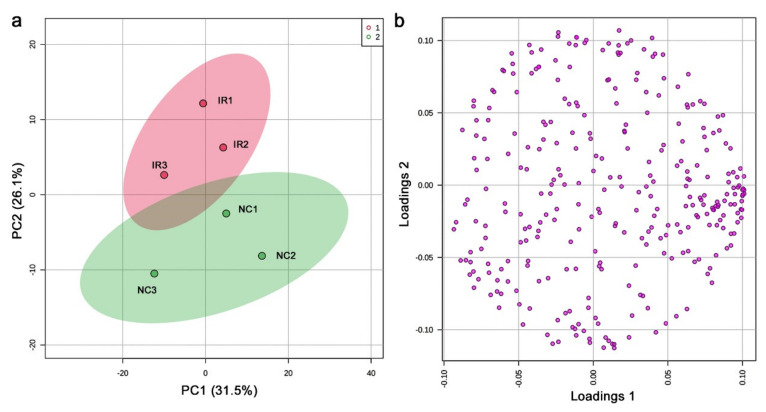
PCA of the nontargeted GC-MS analysis. (**a**) Score plot; 95% confidence ranges are colored. IR, irradiated; NC, nonirradiated. (**b**) Loading plot; 306 peaks are located in a 2D plane.

In the nontargeted GC-MS method, 456 peaks were originally detected, including those detected only in a single sample. The shortest retention was 7.052 min, and the longest retention time was 63.095 min. Among them, 306 peaks were considered valid by MetaboAnalyst. We then performed PCA. PC1 and PC2 explained only 31.5% and 26.1% of the variance, respectively (Figure 7a). Notably, the irradiated and nonirradiated groups clustered individually, keeping the relative positions of the three samples intact. A likely interpretation of these data is that the nonirradiated samples in the negative PC2 area shifted up on the PC2 axis upon irradiation to be placed in the positive PC2 area as the irradiated samples. In other words, irradiation may be a major contributor to PC2, suggesting that a systematic change to the plant metabolites might have been caused by irradiation exposure. The loading plot showed that the peaks were scattered throughout, and some were clustered at the high PC1 region but not at the high PC2 region (Figure 7b), making a biological interpretation of the loading plot from the viewpoint of irradiation difficult.

**Figure 8:**
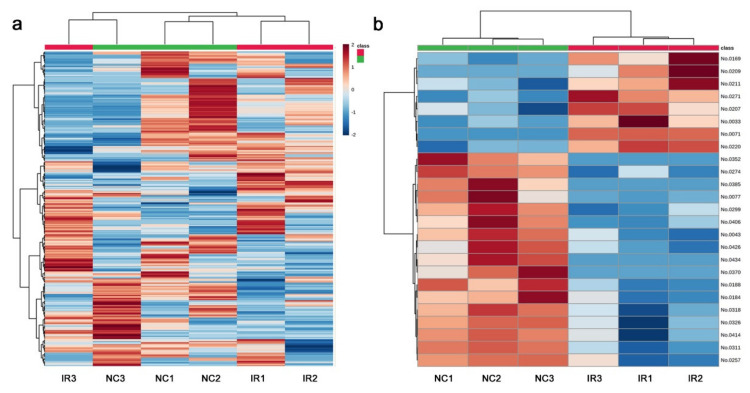
Heat maps from the nontargeted GC-MS analysis. At the top, the irradiated group was indicated by red horizontal bars, and the nonirradiated control group was indicated by green horizontal bars. (**a**) Heat map using all 306 peaks. (**b**) Heat map using the top 25 peaks.

The heat map with all 306 peaks was too complex to decipher any legitimate pattern (Figure 8a), but a heat map with the top 25 peaks indicated differences in the metabolite levels between the irradiated and nonirradiated groups (Figure 8b). The three irradiated samples had a similar pattern to one another, and the three nonirradiated control samples also had a similar pattern to one another, which was different from that of the irradiated group. In other words, a systematic response to the irradiation treatment was observed in all the samples, which was consistent with the PCA results. As in the targeted analysis above, the nonirradiated group appeared to have more metabolites present at higher levels than the irradiated group, suggesting that irradiation might have caused an overall metabolic slowdown in this plant, at least as determined by the nontargeted GC-MS method.

**Figure 9:**
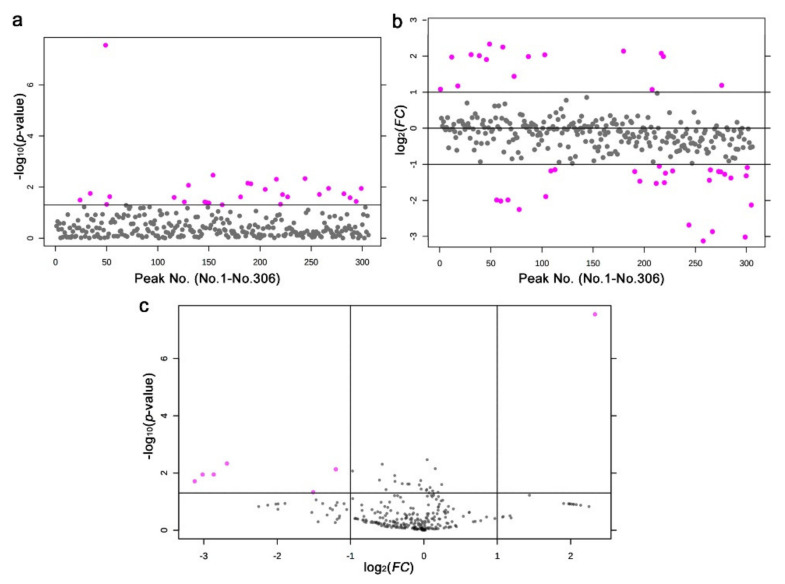
Results of the *t*-test and fold change analysis of the nontargeted GC-MS analysis between the irradiated and nonirradiated groups. (**a**) Distribution of the *p*-values (Student’s *t*-test). The threshold was set at *p* = 0.05. The 28 pink dots indicate significant peaks. The peak numbers (No. 1–No. 306) here on the horizontal axis do not correspond to the original peak numbers shown in Figure 10, because the original peak numbers were assigned before elimination of the nonsense peaks by MetaboAnalyst (also in (**b**)). (**b**) Distribution of the *FC* values. The thresholds were set at *FC* = 2.0 and *FC* = −2.0. The 16 and 27 pink dots indicate peak upregulation and downregulation by more than twofold, respectively. (**c**) Volcano plot. The 7 pink dots indicate peaks that satisfy both the *p*-value and *FC* value criteria.

We detected 28 significantly different peaks between the irradiated and nonirradiated groups using a *t*-test (*p* < 0.05) (Figure 9a); seven peaks had *p* < 0.01, and one had *p* < 0.001. Similarly, we performed a fold change analysis, in which 16 peaks were detected as *FC* > 2.0 and 27 peaks as *FC* < −2.0 (Figure 9b). It appears that more samples were downregulated (negative *FC* values) than upregulated (positive *FC* values), which was consistent with the results of the heat map above. Among them, seven peaks satisfied both conditions (*p* < 0.05 and *FC* > 2.0 or *FC* < −2.0) (Figure 9c).

**Figure 10:**
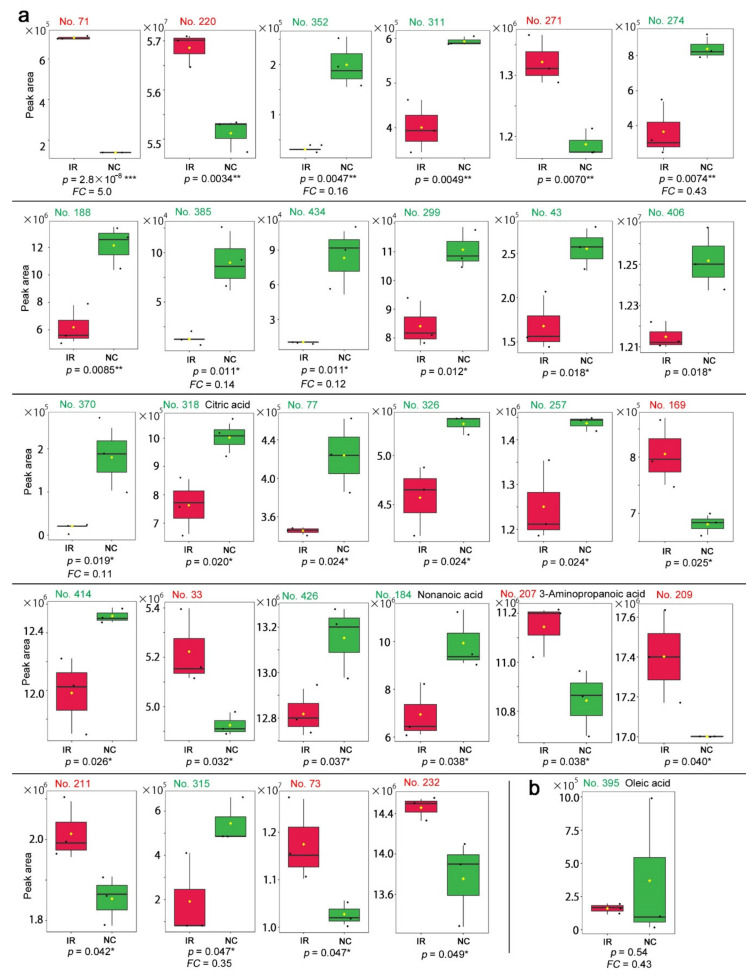
Box plots of the peaks in the nontargeted GC-MS. The irradiated group (IR) is indicated by red boxes, and the nonirradiated control group (NC) is indicated by green boxes. The peak numbers are shown in red when upregulated, and they are shown in green when downregulated. (**a**) Peaks with significant differences between the irradiated and nonirradiated groups in the order of low-to-high *p*-values. There were 28 peaks with *p* < 0.05 (Student’s *t*-test), and among them, only 3 peaks were annotated. The *FC* values are shown when *FC* > 2.0 or *FC* < 0.5. (**b**) Peak No. 395 identified by the fold change analysis. This peak was annotated as oleic acid. * *p* < 0.05, ** *p* < 0.01, *** *p* < 0.001.

Among the peaks with *p* < 0.05, 10 peaks were upregulated upon irradiation, whereas 18 peaks were downregulated (Figure 10a). Only three peaks were annotated as follows: citric acid (No. 318, *p* = 0.020), nonanoic acid (No. 184, *p* = 0.038), and 3-aminopropanoic acid (No. 207, *p* = 0.038). The latter two were also found in the targeted method above. Citric acid and nonanoic acid were downregulated, whereas 3-aminopropanoic acid was upregulated in contrast to its downregulation in the targeted method, questioning the validity of this result.

Among the peaks with *FC* > 2.0 or *FC* < −2.0, 16 peaks were detected as upregulated and 27 as downregulated. However, among them, only one peak was annotated as oleic acid (No. 395) (Figure 10b). Oleic acid was also detected in the targeted method with *FC* < −2.0 (i.e., *FC* < 0.5), but its box plot in both methods suggested that this difference was dependent on a single outstanding value (Figure 6b and Figure 10b), questioning the validity of this result.

Oxalic acid was detected in a large amount as peak No. 76 from the nontargeted method, and its peak levels were not significantly different between the irradiated and nonirradiated groups (*p* = 0.67; Student’s *t*-test).

### 3.4. LC-MS

**Figure 11:**
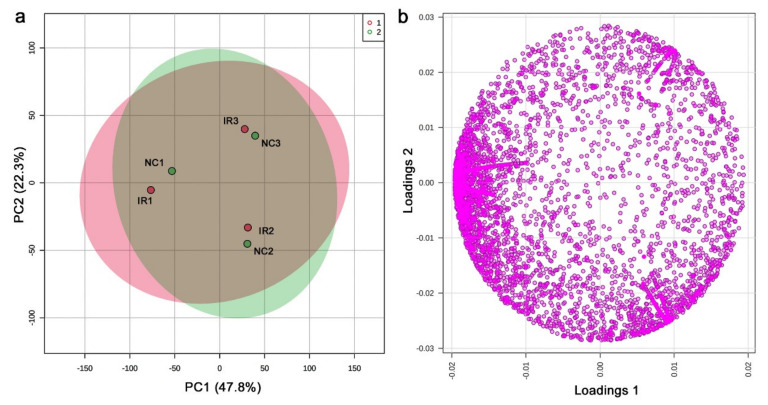
PCA results from the LC-MS analysis. (**a**) Score plot; 95% confidence ranges are colored. IR, irradiated; NC, nonirradiated. (**b**) Loading plot; 5418 peaks are located in a 2D plane.

From the LC-MS analysis, 9554 peaks were originally detected, including those detected in only a single sample; the shortest retention time was 3.001 min (C_9_H_16_O_5_N_2_), and the longest retention time was 24.996 min (C_43_H_66_O_14_). Among them, 5418 peaks were considered valid by MetaboAnalyst. Based on the peak area (intensity) data, the PCA was performed (Figure 11a). PC1 and PC2 explained 47.8% and 22.3% of the variance, respectively. Irradiated and nonirradiated samples from the same plant individual were located close to each other, suggesting that the coordinates of the samples were determined mainly by genetic background and marginally by radiation response. The loading plot showed that there were many peaks in the negative area of the horizontal axis (Figure 11b), but the biological interpretation was difficult.

**Figure 12:**
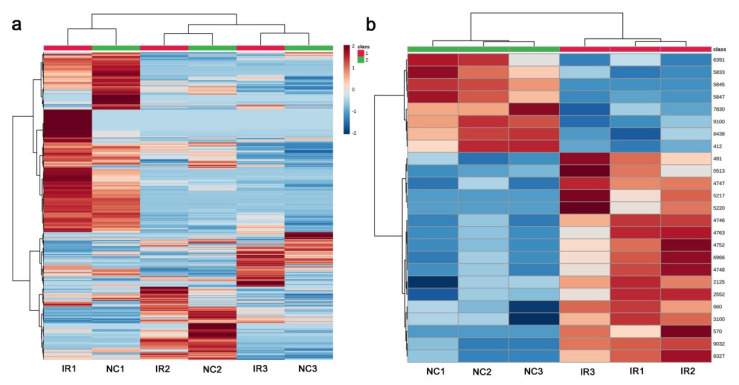
Heat maps from the LC-MS analysis. At the top, the irradiated group was indicated by red horizontal bars, and the nonirradiated control group was indicated by green horizontal bars. (**a**) Heat map using all 5418 peaks. (**b**) Heatmap using the top 25 peaks.

A heatmap of all 5418 peaks also demonstrated that a clonal pair of irradiated and nonirradiated samples of the same plant exhibited similar patterns (Figure 12a), suggesting that genetic differences contributed more substantially to their metabolite differences. However, when the top 25 peaks were examined, the irradiated and nonirradiated groups showed different patterns, and the irradiated group appeared to have more samples with higher metabolite levels than the nonirradiated group (Figure 12b). This result was different from that of GC-MS.

**Figure 13:**
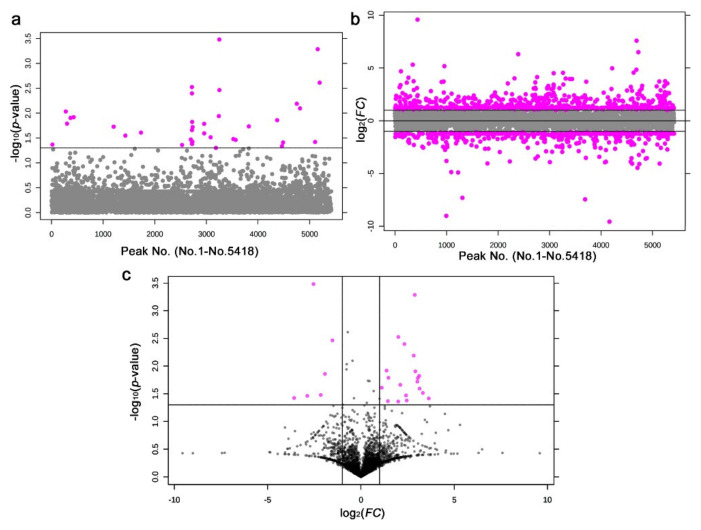
Results of the *t*-test and fold change analysis after the LC-MS analysis between the irradiated and nonirradiated groups. (**a**) Distribution of the *p*-values (Student’s *t*-test). The threshold was set at *p* = 0.05. The 36 pink dots indicate significant peaks. The peak numbers (No. 1–No. 5418) here on the horizontal axis do not correspond to the original peak numbers shown in Figure 14, because the original peak numbers were assigned before elimination of the nonsense peaks by MetaboAnalyst (also in (**b**)). (**b**) Distribution of the *FC* values. The thresholds were set at *FC* = 2.0 and *FC* = −2.0. The 902 and 547 pink dots indicate peaks that were upregulated and downregulated more than twofold, respectively. (**c**) Volcano plot. The 25 pink dots indicate peaks that satisfy both the *p*-value and *FC* value criteria.

We detected 36 significantly different peaks between the irradiated and nonirradiated samples using *t*-tests (*p* < 0.05) (Figure 13a). Similarly, we also performed a fold change analysis (Figure 13b). We found 902 upregulated and 547 downregulated peaks. Since there were too many peaks to investigate, they were not pursued further. Notably, 25 peaks satisfied both criteria (*p* < 0.05 and *FC* > 2.0 or *FC* < −2.0) (Figure 13c).

**Figure 14:**
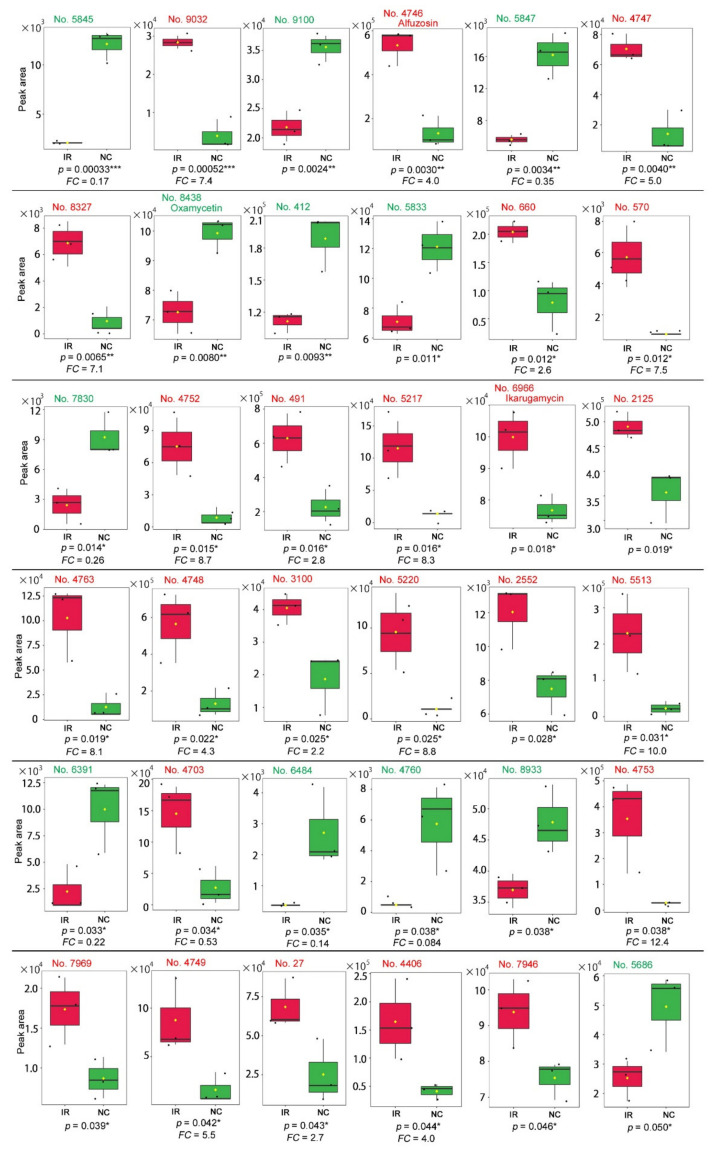
Box plots of the 36 peaks detected by the LC-MS analysis. The irradiated group (IR) is indicated by red boxes, and the nonirradiated control group (NC) is indicated by green boxes. The peak numbers are shown in red when upregulated, and they are shown in green when downregulated. Peaks with significant differences between the irradiated and nonirradiated groups (*p* < 0.05, Student’s *t*-test) are shown in the order of low-to-high *p*-values. Among them, only 3 peaks are singularly annotated. The *FC* values are shown when *FC* > 2.0 or *FC* < 0.5. * *p* < 0.05, ** *p* < 0.01, *** *p* < 0.001.

Among the peaks with *p* < 0.05, 24 peaks were upregulated after irradiation, whereas 12 peaks were downregulated (Figure 14). Most peaks had no compound name annotations, whereas some were annotated as multiple compounds. Only three peaks were annotated as a single compound as follows: alfuzosin (No. 4746, *p* = 0.0033), oxamicetin (oxamycetin) (No. 8438, *p* = 0.0080), and ikarugamycin (No. 6966, *p* = 0.018).

### 3.5. Functional Categorization of the LC-MS Annotated Peaks

There were 12 peaks with multiple annotations and three peaks with a single annotation (Table 1). We checked their MS/MS fragment spectrograms, but similar ones were not found in the searched databases (Supplementary Table S2; MS/MS spectrograms can be seen by double-clicking the peak number in the MS2 tab in this Microsoft Excel file). These 15 peaks were classified into four functional categories (Table 2). The first functional category was “antioxidation”, in which plant-derived antioxidants were included. These compounds were all upregulated upon irradiation. No. 660 (*p* = 0.012) was identified as either L-L-homoglutathione or S-methylglutathione. No. 2125 (*p* = 0.019), and No. 4406 (*p* = 0.044) had multiple candidates, but they were flavonoids, which is a group of phytochemical antioxidants [63].

The second functional category was the “stress response”, in which plant-derived compounds for the stress resistance were included. All of these compounds, except one, were upregulated upon irradiation. No. 4746 (*p* = 0.0030) was alfuzosin, which is known to have pharmacological activity in animals [64,65,66,67]. No. 4763 (*p* = 0.019) and No. 2552 (*p* = 0.028) had multiple candidates, partly due to their small masses, but the candidates for No. 4763 and No. 2552 included dihydrobenzofuran (coumaran) and 2-acetylfuran, respectively. The former is well-known as a natural biopesticide [68]. No. 7969 (*p* = 0.039) had multiple candidates, but they were all alkaloids. No. 4753 (*p* = 0.038) was identified as either 7-chlorodeutziol or myobontioside A. These compounds belonged to a group of iridoids, which are plant chemicals that probably ward off insects and other animals [69,70]. No. 7830 (*p* = 0.014) had five (virtually three due to redundancy) candidate compounds that were all plant-derived secondary metabolites. One of them was a limonoid, a possible antifeedant [71], and another was a taxoid [72]. However, No. 7830 was downregulated.

The third functional category was “non-plant derivatives”. All of these compounds, except one, were downregulated upon irradiation, probably because of the sterilization effect of irradiation. This plant sample might have been contaminated by microorganisms despite the washing process of the collected leaves. No. 8438 (*p* = 0.0080) and No. 6966 (*p* = 0.018) were identified as oxamicetin (oxamycetin) and ikarugamycin, respectively, and are known to be antifungal antibiotics produced by a group of soil bacteria, *Streptomyces* [73,74,75]. No. 412 (*p* = 0.0093) was identified as either leinamycin or uracil, and if the former is correct, this peak is another antibiotic produced by the same soil bacterial genus *Streptomyces* [76]. No. 5833 (*p* = 0.011) had three candidate compounds, all of which were mycotoxins from fungi [77,78]. The upregulation of No. 6966, annotated as ikarugamycin, was enigmatic, but a group of soil bacteria might have responded to the irradiation treatment.

The fourth functional category was simply “unknown”. No. 4703 (*p* = 0.034) and No. 8933 (*p* = 0.038), included in this category, had various candidate compounds.

### 3.6. LC-MS/MS Spectrograms of Peak No. 4746 and Alfuzosin

**Figure 15:**
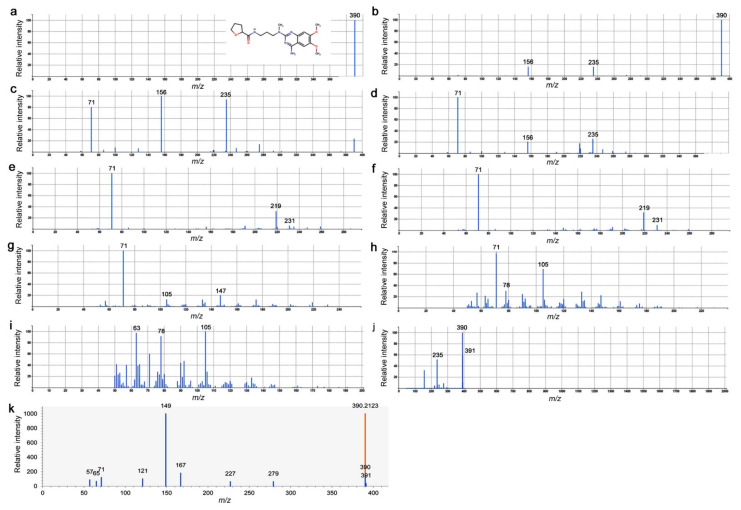
LC-MS/MS spectrograms of alfuzosin and peak No. 4746 (from the present study) after positive ionization ([M+H]^+^). (**a**–**j**) Ten experimental HMDB records of alfuzosin. The highest intensity was set as 100, and the relative intensity values of the fragments are shown. The top 3 fragments are labeled according to their *m/z* values in each panel. The inset in (**a**) is the structure of alfuzosin, in which oxygen and nitrogen atoms and bonds are shown in red and purple, respectively. The splash keys for the identification of these spectrograms from (**a**) to (**j**) (after “splash10-“) are as follows: 0006-0009000000-58fd75abdf178ea1b331, 0006-0119000000-90a7799e494035d224e7, 0abi-5791000000-648d5529faeac9e35dc0, 00di-9260000000-591f2bacf7d82aeba7e3, 00di-9250000000-d107a0e9675604325 bb1, 00di-9340000000-f708562f3998488d7fce, 00di-7910000000-3fe44ce2a42f727569e2, 05fr-8900000000-dca38c682ad0 b6c90b46, 0bvl-9300000000- bad5c36d48e0e93a82aa, and 0006-0249000000-93552ff8c72ff220f172. (**k**) Present study. The major fragments are labeled by *m/z* values. The full records of the ionized fragments [M+H]^+^ (*m/z*) and their relative intensity values (in parenthesis; the highest intensity was set as 1000) are as follows: 390.212 (1,000) (shown in brown), 149.02 (999), 167.03 (179), 390.21 (152), 71.09 (120), 121.03 (100), 57.07 (87), 65.04 (66), 227.13 (62), 279.16 (62), 391.19 (38), 93.07 (36), 67.62 (32), 113.13 (17), 391.08 (17), 121.04 (17), 183.10 (16), 93.03 (16), 107.09 (16), 390.16 (14), 79.05 (13), 163.11 (13), 228.13 (13), 209.12 (13), 169.09 (12), 137.06 (12), 95.05 (11), 111.04 (11), 81.07 (11), 119.09 (10), 390.14 (10), 365.63 (10), 413.48 (10), 351.38 (10), 255.77 (9), 184.48 (9), 364.65 (9), 304.16 (9), 354.17 (8), 105.07 (8), 81.03 (8), 55.06 (8), 220.88 (8), 153.82 (7), 109.22 (7), 53.76 (7), and 91.48 (7). The fragment at 228.13 (*m/z*) indicates a loss of 162.08, suggesting the loss of a hexose moiety (C_6_H_10_O_5_).

As mentioned above, peak No. 4746 was singularly annotated as alfuzosin. Alfuzosin is a synthetic quinazoline derivative with an exact mass of 389.206. Since alfuzosin is not a plant-derived drug, peak No. 4746 should not be identical to alfuzosin. Peak No. 4746 was estimated to have a very similar mass; its *m/z* value for [M+H]^+^ was 390.214. However, automated annotation does not guarantee chemical identification. The difference between the theoretical and experimental exact mass values (dPPM value) was calculated to be −3.37 ppm, which was acceptable for annotation but relatively large in absolute values among the annotated peaks. Here, we examined the LC-MS/MS spectrograms of peak No. 4746 and compared them to those of alfuzosin recorded in the Human Metabolome Database (HMDB) [60,61] (Figure 15).

Ten experimental [M+H]^+^ spectrograms of alfuzosin revealed that the major fragments of alfuzosin were located at 235, 156, and 71 (*m/z*), and further fragmentation produced peaks with 231, 219, 147, 105, 78, and 63 (*m/z*) (Figure 15a–j). In contrast, the major fragments of No. 4746 in the present analysis were located at 279, 227, 167, 149, 121, 71, 65, and 57 (*m/z*); among which, the fragment at 149 (*m/z*) had the highest intensity (Figure 15k). Between the experimental records and the present analysis, only the fragment at 71 (*m/z*) was identical. The fragment at 149 (*m/z*) in the present analysis was close to the fragment at 147 (*m/z*) in the database, but their relative intensity values were different. Furthermore, one of the fragments from peak No. 4746 was predicted to be C_6_H_10_O_5_. This means that No. 4746 had a hexose moiety, which was not found in alfuzosin. Taken together, it is likely that peak No. 4746, annotated as alfuzosin, was not actually alfuzosin itself but, instead, an unknown structural isomer of alfuzosin or an unknown compound similar to alfuzosin.

## 4. Discussion

### 4.1. Experimental System

In this study, we examined the metabolomic response of the creeping wood sorrel, the host plant of the pale grass blue butterfly, to low-dose radiation exposure. To reproduce the possible radiation environment of Fukushima, contaminated soil was collected from Fukushima, and plants collected from Okinawa, the least affected prefecture in Japan, were irradiated to exclude a history of previous exposure. We believe that this experimental system uniquely provided us with information on the acute perturbation of the plant in Fukushima immediately after the nuclear accident. Additionally, we may be able to obtain a hint at the ongoing chronic impacts of the Fukushima nuclear accident on the ecosystem. For accurate understanding of the chronic impacts, metabolomic analyses of field-collected plants from Fukushima should be performed. Having mentioned this point, we clarified that the major radionuclide in the soil used in the present study was anthropogenic ^137^Cs, as expected.

The plant looked completely healthy after irradiation. In fact, the cumulative absorbed dose and dose rate in the creeping wood sorrel in this system were understood as “very low” compared with other low-dose (rate) experiments. For example, one experiment employed 185.4 mGy/h and 1.1 Gy for the low-dose exposure to mice [48], which is 5453-fold and 193-fold higher than in the present study, respectively. The external exposure experiment using the pale grass blue butterfly and its host plant in the original paper [19] was estimated to be 55 mSv and 125 mSv. These values are 9.6-fold and 22-fold higher than in the present study (5.7 mGy), assuming that Sv and Gy are interchangeable. Notable exceptions were in situ exposure experiments at Iitate Village, where the exposure level was 4 μSv/h for 3 days [15,17].

Considering the very low exposure level in the present study, it is not surprising that only a small number of significantly different peaks were detected by the GC-MS and LC-MS analyses. Rather, it may be surprising that the plant nonetheless responded metabolically to 5.7 mGy of irradiation treatment. We primarily used *p* < 0.05 as the criterion for differentially expressed peaks. This relatively generous criterion was unavoidable for this study to gain a substantial amount of information on the differentially expressed metabolites under very low exposure levels. Accordingly, at least some candidate peaks may be false positives. This problem may partially be resolved by increasing the number of samples. Another difficulty of this study was that the information in the databases on the secondary metabolites of this non-model organism appeared to be limited. Below, with these limitations in mind, we discuss the present results.

### 4.2. Interpretations of the GC-MS Results

In both the targeted and nontargeted GC-MS methods, the irradiated and nonirradiated groups showed different peak distribution patterns. It appears that, by GC-MS, the irradiated group showed the overall lower peak intensity values, which were clearly seen in the fold change distributions (Figure 5b and Figure 9b). The PCA of the targeted method (Figure 3) showed that the irradiated group clustered in a much narrower range than the nonirradiated control group. The wider distribution of the nonirradiated group could be interpreted as the genetic difference, and the wider distribution was made narrower after irradiation, suggesting a stereotypical response. Similarly, the PCA of the nontargeted method (Figure 7) showed a systematic upward shift upon irradiation along PC2. In both cases, it is likely that the plant indeed responded to this level of irradiation systematically. In the nontargeted method, PC2 (26.1%) may represent the irradiation effects, and in contrast, PC1 (31.5%) may represent the possible genetic effects. The contribution of PC1 was larger than that of PC2, but they may be considered comparable. The peaks identified by the fold change analysis revealed that they were all downregulated, and their distribution ranges became much narrower in response to irradiation, being consistent with the PCA results. A rough interpretation could be that metabolomic changes of a group of plant chemicals in response to irradiation at the milligray level were as large as the genetic differences among the plant species in Okinawa.

We were able to examine box plots of each candidate peak from the GC-MS. From the targeted method, the peaks annotated as caproic acid, nonanoic acid, and 3-aminopropanoic acid were downregulated, whereas the peak annotated as lauric acid was upregulated (*t*-test). Caproic acid, also known as hexanoic acid, has been shown to be an inducer of plant defense mechanisms [79]. This function of caproic acid is different from, but similar to, that of azelaic acid [79], another candidate (fold change analysis). Moreover, azelaic acid is a derivative of oleic acid [79], which is yet another candidate (fold change analysis). Together, it can be speculated that their metabolic pathways and defense functions may be perturbed by irradiation. Nonanoic acid is known as a phytotoxic chemical that may function to eradicate other neighboring plants [80]. Lauric acid can function as an antifungal and possible antiviral agent [81]. It has been speculated that lauric acid is upregulated as a part of the plant defense mechanism under irradiation stress.

A carbohydrate pathway may be slowed down, since glucose and fructose may be downregulated (fold change analysis), as observed from the results of the targeted method. This is consistent with the finding that citric acid (*p* = 0.020; *t*-test) was also found to be downregulated by the nontargeted method, suggesting that the TCA cycle may be slowed down. Alternatively, a photosynthetic pathway that produces glucose and fructose may be slowed down. Nonanoic acid was found in not only the targeted method but, also, the nontargeted method (*t*-test). Oleic acid was also found in the nontargeted method (fold change analysis), although this result is not credible based on its box plot (Figure 10b) because of a single outstanding value. 3-Aminopropanoic acid, also known as β-alanine, is a compound related to the general stress response in plants [82] and was found by both the targeted and nontargeted methods (*t*-test) with different results; it was upregulated in the former and downregulated in the latter. This was the only case in which the targeted and nontargeted results contradicted each other. Since the targeted method is more reliable than the nontargeted method in terms of the alignment and annotation, the latter could simply be an annotation mistake. If so, the upregulation of 3-aminopropanoic acid could indicate the activation of a defense mechanism.

Notably, many peaks were not annotated in the nontargeted method, but some were highly significant. The most significant peak from the GC-MS analysis was No. 71 (*p* = 2.8 × 10^−8^), which showed a marked upregulation (*FC* = 5.0) (Figure 10a). We confirmed that oxalic acid was detected at a high level in both the irradiated and nonirradiated groups with no statistically significant differences in either the targeted or nontargeted methods. Even if the oxalic acid in the leaves is a feeding stimulant for larvae of the pale grass blue butterfly [62], irradiated leaves would not cause a feeding problem in terms of the level of oxalic acid.

### 4.3. Interpretations of the LC-MS Results

The LC–MS PCA results indicated that irradiated and nonirradiated samples of the same clone were located more closely together in the score plot than the irradiated or nonirradiated groups (i.e., the same treatment group). (Figure 11). This result was in contrast to the GC-MS results, in which a systematic change in the irradiated group along PC2 was observed. Furthermore, by LC-MS, the number of upregulated significant peaks was greater than the number of downregulated peaks (Figure 13b), which was also in contrast to the GC-MS results. An interpretation of these data is that the downregulation of a group of GC-MS-detected metabolites (including many primary metabolites) upon irradiation is a relatively stereotypical response in this plant, but the upregulation of a group of LC-MS-detected metabolites (including many secondary metabolites) upon irradiation is more dependent on the genetic background of the individual plant. In other words, the response profiles may be stereotyped but simultaneously allow individual variations. To examine by PCA if there are any systematic irradiation responses to the low-dose levels, GC-MS seems to be superior to LC-MS. On the other hand, to examine by *t*-test if there are any upregulated peaks, LC-MS seems to be more suitable than GC-MS.

A limited number of peaks were annotated, but the plants appeared to upregulate the production of antioxidants and certain chemicals important for stress resistance and defense, including potential natural insecticides (Table 2). We consider that these two functional categories represent reasonable mechanistic response profiles of this plant. How plants receive irradiation signals is not known, but reactive oxygen species (ROS) may be produced inside cells, which may activate a series of general stress responses. The antioxidants detected here may play a role in scavenging these ROS. Interestingly, it seems that not all of the potential defense metabolites were upregulated. As discussed before, most compounds for the defense mechanisms, such as caproic acid, were downregulated, whereas lauric acid was upregulated in GC-MS. Similarly, in LC-MS, most of the annotated peaks in the second functional category were upregulated, but one was downregulated, which was likely a limonoid. It appears that there are many defense pathways, and some pathways may be activated more often than others in response to low-level radiation stress. Similarly, only a limited number of potential antioxidants were upregulated in the present study.

Perhaps because of this stress response or because of the direct radiation sterilization, non-plant derivatives such as oxamicetin (*p* = 0.0080), which may be contaminants from soil microorganisms, tended to be downregulated in the irradiated group (Figure 14). Additionally, ikarugamycin (*p* = 0.018), which may also be a contaminant, was upregulated. These annotations may be due to coincidences in the exact mass values, but the positive involvement of soil microbes, including endophytic bacteria and fungi, during the radiation response of the plant cannot be ruled out.

Peak No. 4746 was singularly annotated as alfuzosin with a reasonably small *p*-value (*p* = 0.0030) and high *FC* value (*FC* = 4.0) (Figure 14). In reference to the HMDB LC-MS/MS spectrogram records, peak No. 4746 was not alfuzosin itself but, instead, an unknown isomer that may contain a hexose moiety (Figure 15). Thus, the alfuzosin annotation for peak No. 4746 may be considered just a coincidence. Nonetheless, a functional coincidence may follow if their masses and affinities detected by LC-MS were identical. It is noteworthy that alfuzosin is a pharmaceutical drug that blocks α_1_-adrenergic receptors in humans [64,65,66,67]. Considering that biogenic amines such as tyramine and octopamine play important physiological roles in insects similar to adrenergic transmitters in vertebrates [83,84,85,86], it may be possible that this unknown alfuzosin isomer in the plant functions as an antagonist of biogenic amine receptors after ingestion by insects to cause pharmacological effects on insect feeding behaviors and the metabolism.

We also found other unannotated plant derivatives that were significantly different between the irradiated and nonirradiated groups. The lowest *p*-value was assigned to peak No. 5845 (*p* = 3.3 × 10^−4^) with downregulation (*FC* = 0.17) (Figure 14). The peak with the second-lowest *p*-value was No. 9032 (*p* = 5.2 × 10^−4^) with upregulation (*FC* = 7.4) (Figure 14). Unfortunately, their structural identities are unknown.

### 4.4. Ecological Field Effects

Here, we found metabolomic changes in the investigated plant in response to very low levels of radiation exposure. We think that the detected upregulated or downregulated metabolites with or without annotation together might have contributed to the high mortality and morphological abnormality rates of the pale grass blue butterfly reported previously. In other words, changing the levels of the leaf metabolites may be an important part of the mechanism of the ecological field effects that were caused by the Fukushima nuclear accident. It should also be tested whether the pale grass blue butterfly responds to this radiation source (i.e., contaminated soil from Fukushima) physiologically or morphologically when reared together with the host plant.

Additionally, other ecological field effect modes of action may also be valid and important [27,32]. For example, a sodium deficiency is probably an important mechanism of the ecological field effects [38]. Another possibility in the plant is a decrease in their levels of vitamins available for the larvae when irradiated. Although some vitamins were found in the list of annotated compounds in the present study, no statistically significant difference was observed between the irradiated and nonirradiated groups. Furthermore, the biological impacts of the Fukushima nuclear accident include transgenerational effects caused by the initial high-dose exposure [19,34,36].

## 5. Conclusions

This study revealed that the creeping wood sorrel *Oxalis corniculata* responded at the metabolome level to low-level radiation exposure from soil contaminated by the Fukushima nuclear accident. Upon irradiation, the plant may reduce the levels of some compounds in carbohydrate metabolic pathways and some stress-related or defense mechanisms while simultaneously increasing the levels of some secondary metabolites that function as antioxidants and stress-related or defense chemicals. Some plant chemicals, including the discovered alfuzosin isomer, might have contributed to the high rates of mortality and abnormality observed among the butterflies in the field. Taken together, the results of this study demonstrated the metabolomic response of this *Oxalis* plant to low-dose radiation exposure and implicated the potential ecological field effects of low-level radioactive contamination from the Fukushima nuclear accident.

## Figures and Tables

**Table 1:** Summary of the annotated LC-MS peaks.

No.	Formula	Exact Mass	Up/Down	Annotation (Compound Name)
4746	C_19_H_27_O_4_N_5_	389.206	UP	Alfuzosin
8438	C_29_H_42_O_10_N_6_	634.296	DOWN	Oxamicetin; Oxamycetin (produced by *Streptomyces inusitatus*)
412	C_4_H_4_O_2_N_2_	112.027	DOWN	Acetylenedicarboxamide; Acetylene diamide; Aquamycin; Cellocidin; Lenamycin; NSC 38643; NSC 65381 (produced by *Streptomyces* sp.); Uracil
5833	C_17_H_22_O_7_	338.137	DOWN	Acetyldeoxynivalenol (a mycotoxin from *Fusarium graminearum*); O-Acetylcyclocalopin A (produced by the mushroom *Boletus calopus*); 15-Acetyl-4-deoxynivalenol (a mycotoxin from *Fusarium graminearum*)
660	C_11_H_19_O_6_N_3_S	321.099	UP	L-L-Homoglutathione (an antioxidant); S-Methylglutathione (an antioxidant)
7830	C_37_H_46_O_12_	682.299	DOWN	Plant-derived pharmacological compounds (*1)
6966	C_29_H_38_O_4_N_2_	478.283	UP	Ikarugamycin (produced by *Streptomyces phaeochromogenes*)
2125	C_15_H_18_O_11_	374.085	UP	Flavonoids (*2)
4763	C_8_H_8_O	120.058	UP	Various compounds (*3) including Dihydrobenzofuran in plants such as *Lantana camara* as a natural biopesticide
2552	C_6_H_6_O_2_	110.037	UP	Various compounds (*4) including 2-Acetylfuran in plants
4703	C_10_H_14_O_3_	182.094	UP	Various compounds (*5)
8933	C_13_H_20_O	192.151	DOWN	Various compounds (*6)
4753	C_15_H_23_O_9_Cl	382.103	UP	7-Chlorodeutziol; Myobontioside A (plant derivatives)
7969	C_21_H_23_O_6_N	385.153	UP	Colchicine-related alkaloids (*7)
4406	C_32_H_36_O_18_	708.190	UP	Flavonoids (*8)

*1: 2,7-Dideacetyltaxuspine X; (+)-2,7-Dideacetyltaxuspine X; Swietephragmin C (a limonoid from an African medicinal plant); Taxezopidine K; (+)-Taxezopidine K (isolated and purified from the seeds of the Japanese yew *Taxus cuspidata* and an inhibitor of Ca^2+^-induced depolymerization of microtubules). *2: 6-[5-(2-Carboxy-2-hydroxyethyl)-2-hydroxyphenoxy]-3,4,5-trihydroxyoxane-2-carboxylic acid; 6-{[3-(3,4-Dihydroxyphenyl)-2-hydroxypropanoyl]oxy}-3,4,5-trihydroxyoxane-2-carboxylic acid; 6-[5-(2-Carboxyethyl)-2,3-dihydroxyphenoxy]-3,4,5-trihydroxyoxane-2-carboxylic acid; 3,4,5-Trihydroxy-6-{[3-(3,4,5-trihydroxyphenyl)propanoyl]oxy}oxane-2-carboxylic acid; 6-[4-(2-Carboxyethyl)-2,6-dihydroxyphenoxy]-3,4,5-trihydroxyoxane-2-carboxylic acid; 6-[4-(2-Carboxy-2-hydroxyethyl)-2-hydroxyphenoxy]-3,4,5-trihydroxyoxane-2-carboxylic acid. *3: Phenylacetaldehyde; Styrene oxide; 3-Ethenylphenol; *p*-Methylbenzaldehyde; Dihydrobenzofuran (or Coumaran, an acetylcholinesterase (AChE) inhibitor produced by the leaves of *Lantana camara* as a biopesticide); Lentialexin (produced by a mixed culture of *Lentinus edodes* (Shiitake mushrooms) and *Trichoderma polysporum*); 2-Methylbenzaldehyde; Acetophenone; 3-Methylbenzaldehyde; 4-Vinylphenol. *4: Resorcinol; Pyrocatechol; 2-Acetylfuran (found in coffee, passion fruit, and others); 5-Methyl-2-furancarboxaldehyde; (*E,E*)-2,4-Hexadienedial; Hydroquinone. *5: 4-Ethyl-2,6-dimethoxyphenol; Eupatriol; Bombardolide B; Furfuryl pentanoate; Peperinic acid; *threo*-Anethole glycol; Amyl 2-furoate; Verimol J; Stagonolide E; (-)-Stagonolide E; Isoamyl 2-furoate; Fistupyrone; Annularin D; (+)-Annularin D; Furfuryl isovalerate; 2-Ethoxy-4-(methoxymethyl)phenol; Modiolide B; Bombardolide D; (-)-Bombardolide D; Multiflotriol; Crocusatin I; Sapinofuranone B; 4-Hydroxy-6-isopropyl-3,5-dimethyl-2H-pyran-2-one; GSIR-1; 2-(2-Hydroxy-4-methylphenyl)-1,3-propanediol; 9,10-Dihydroxythymol; 4-(Ethoxymethyl)-2-methoxyphenol; Dihydroconiferyl alcohol. *6: 2,5-Diisopropyl-3-methylphenol; Edulan I; 2,3-Diisopropyl-5-methylphenol; alpha-Damascone; Cycloionone; 2,4-Diisopropyl-3-methylphenol; Vitispirane; Isospirene; γ-Ionone; 4-(2,6,6-Trimethyl-1,3-cyclohexadien-1-yl)-2-butanone; 2,5-Diisopropyl-3-methylphenol; Pseudoionone; 2,6-Diisopropyl-3-methylphenol; (*E*)-5,8-Megastigmadien-4-one; δ-Damascone; 4-(2,6,6-Trimethylcyclohex-1-enyl)but-2-en-4-one; Phenethyl isoamyl ether; (2*E*,4*Z*,7*Z*)-2,4,7-Tridecatrienal; 2,4-Diidopropyl-5-methylphenol; 4-(4-Methyl-3-pentenyl)-3-cyclohexene-1-carboxaldehyde. *7: *N*-Formyl-*N*-deacetylcolchicine (an alkaloid from *Gloriosa superba*); Romucosine H (an alkaloid from *Annona cherimola*); *N*-Methylporphyroxine (a rhoeadine alkaloid); Papaverrubine F (an alkaloid); 3-Demethylcolchicine; 2-Demethylcolchicine; (-)-2-Demethylcolchicine; Glaucamine (an alkaloid); Polycarpine (a marine alkaloid). *8: Patuletin 3-rhamnoside-7-(3’’’,4’’’-diacetylrhamnoside); Patuletin 3-(4’’-acetylrhamnoside)-7-(3’’’-acetylrhamnoside); Patuletin 3,7-bis(3-acetylrhamnoside); Kaempferide 3-rhamnoside-7-(6’’-succinylglucoside); Patuletin 3-(4’’-acetylrhamnoside)-7-(2’’’-acetylrhamnoside).

**Table 2:** Functional categories of the annotated LC-MS peaks.

Category No.	Possible Function	Peak No. (Up/Down)	UP/DOWN (Collective)
1	Antioxidation	660 (UP), 2125 (UP), 4406 (UP)	UP
2	Stress response	4746 (UP), 4763 (UP), 2552 (UP), 7969 (UP), 4753 (UP), 7830 (DOWN)	UP (or DOWN)
3	Non-plant derivatives	8438 (DOWN), 6966 (UP), 412 (DOWN), 5833 (DOWN)	DOWN (or UP)
4	Unknown	4703 (UP), 8933 (DOWN)	UP or DOWN

## Data Availability

The data presented in this study and the source data are available in this article and in the Supplementary Materials.

## Supplementary Materials

The following are available online at https://www.mdpi.com/article/10.3390/life11090990/s1: Supplementary Table S1: GC-MS_Result Kazusa DNA Institute. Supplementary Table S2: LC-MS_Result Kazusa DNA Institute. Supplementary Table S3: GC-MS target data file. Supplementary Table S4: GC-MS nontarget data file. Supplementary Table S5: LC-MS data file.
